# Supplementary material for: Synthetic Route to Conjugated Donor–Acceptor Polymer Brushes via Alternating Copolymerization of Bifunctional Monomers
Source: Polymers (Basel). 2022 Jul 4;14(13):2735. doi: 10.3390/polym14132735 (PMC9268968; doi:10.3390/polym14132735)
Supplement: Supplementary file 1 [file polymers-14-02735-s001.zip › polymers-1790647-supplementary.pdf]

Synthetic route to conjugated donor-acceptor polymer brushes via alternating copolymerization of bifunctional monomers

Anna Grobelny [1], Karolina Lorenc [1], Łucja Skowron [1]and Szczepan Zapotoczny [1,*]

SUPPORTING INFORMATION

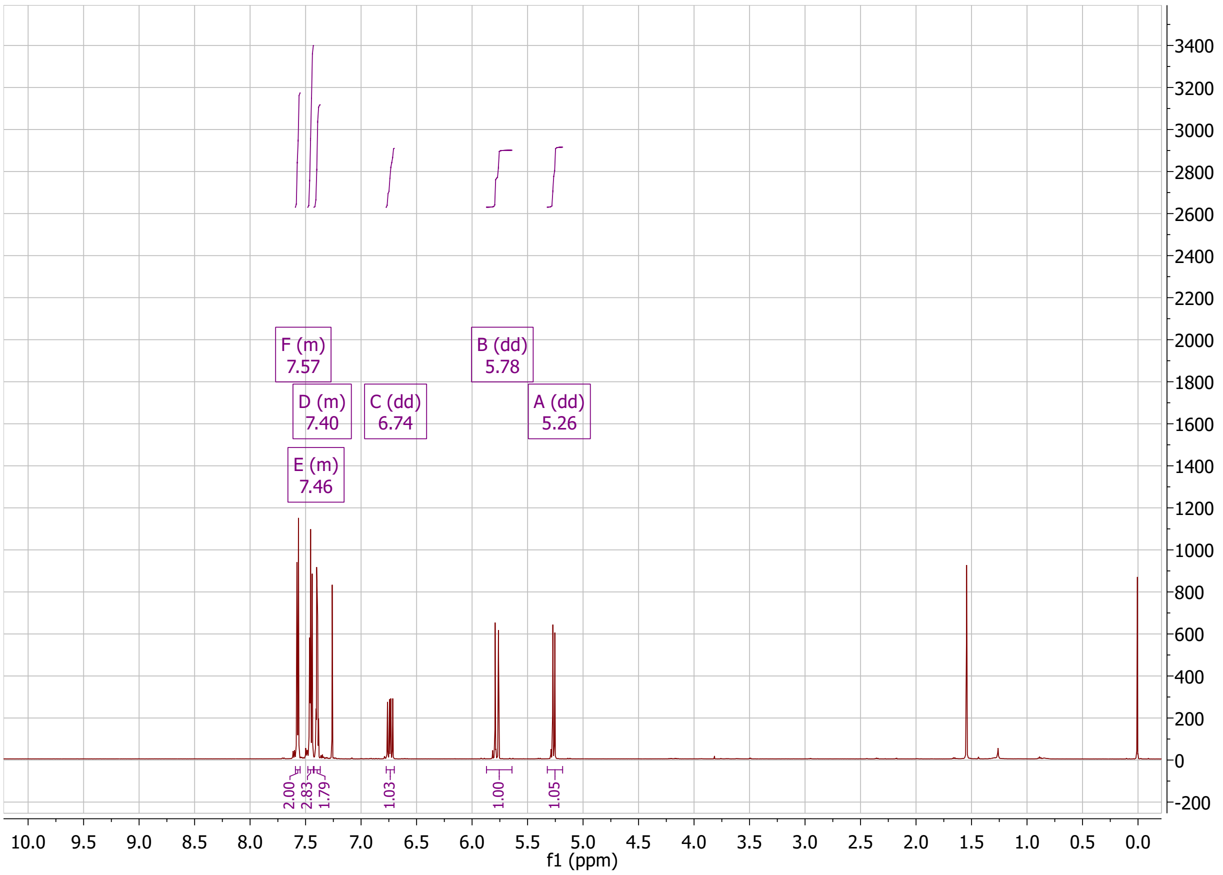

Figure S1. The ^{1}H NMR spectrum of synthesized donor **St-D** monomer.

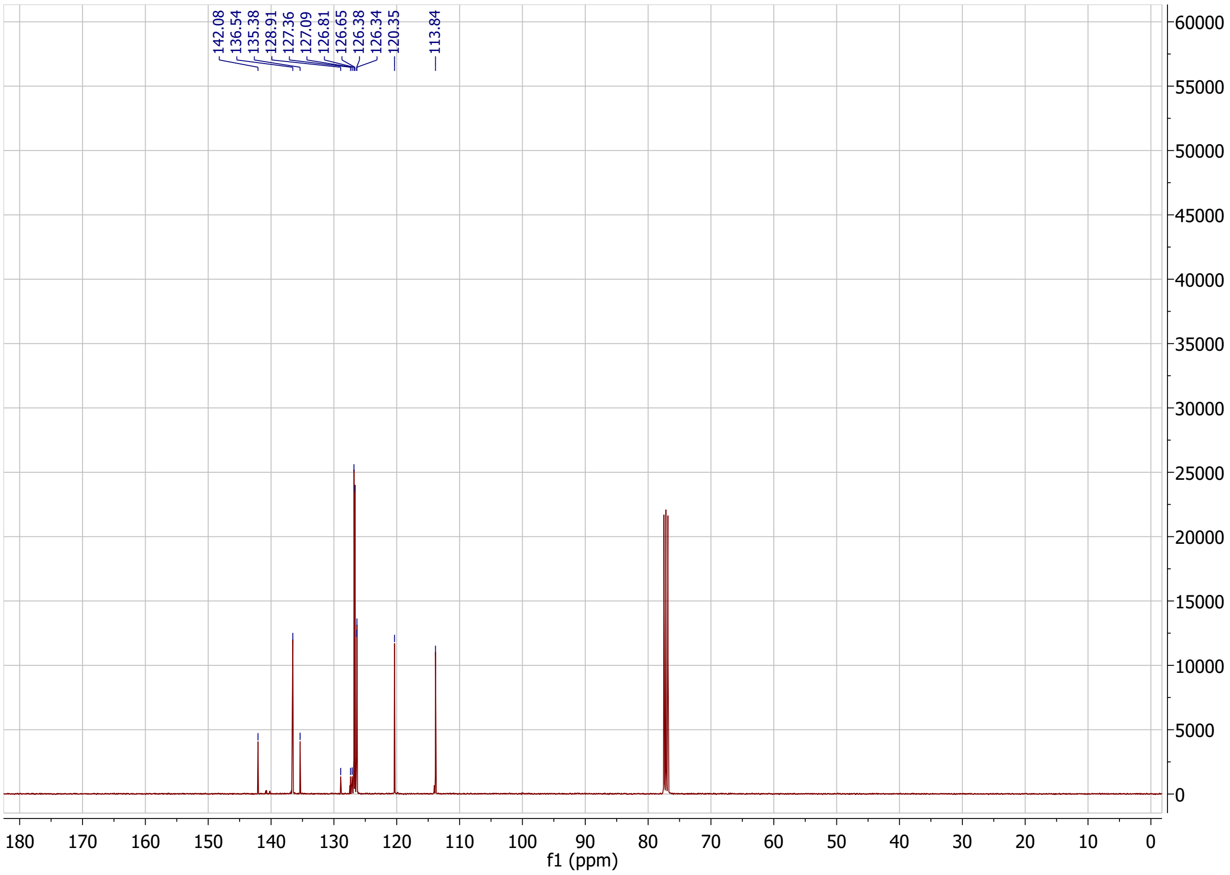

Figure S2. The ^{13}C NMR spectrum of synthesized donor **St-D** monomer.

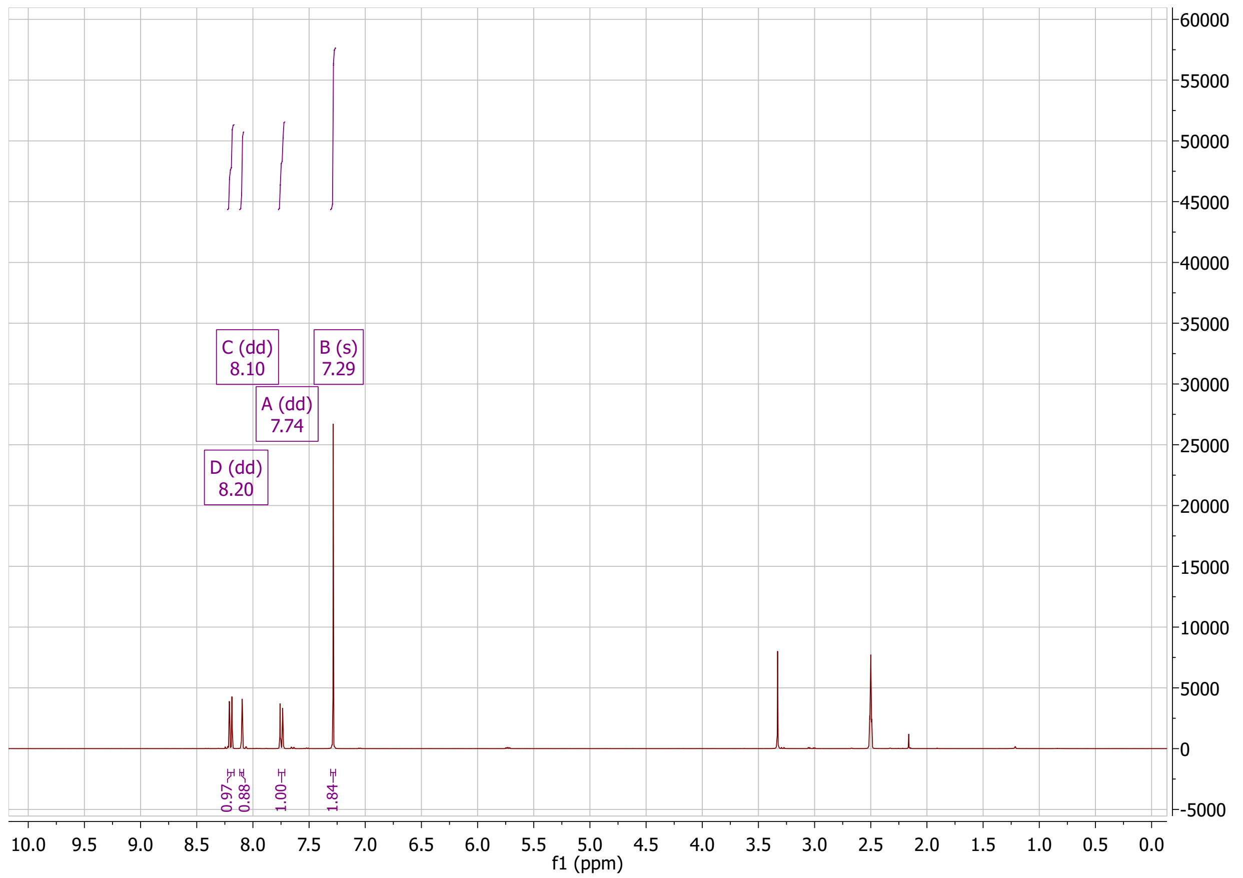

Figure S3. The ^{1}H NMR spectrum of synthesized acceptor **Ma-A** monomer.

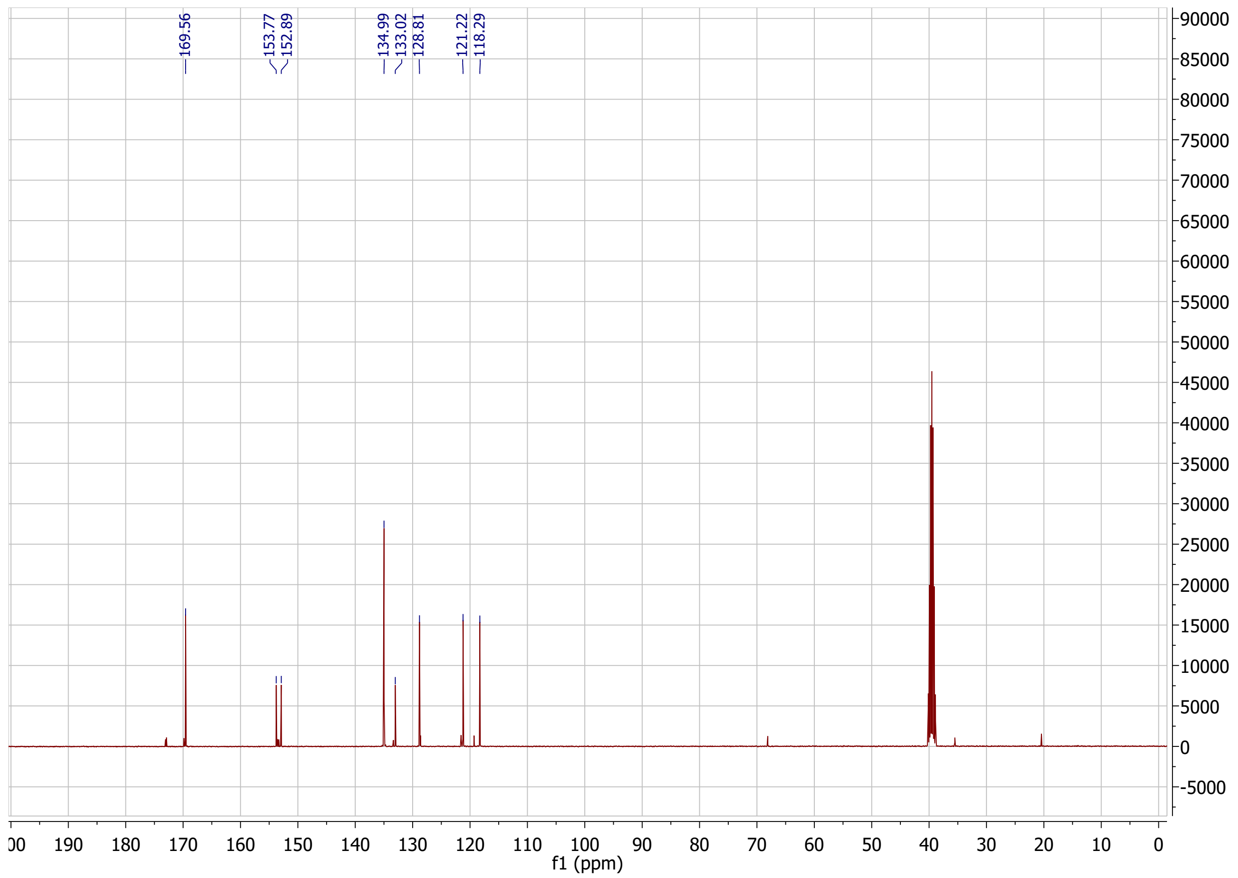

Figure S4. The ^{13}C NMR spectrum of synthesized acceptor **Ma-A** monomer.

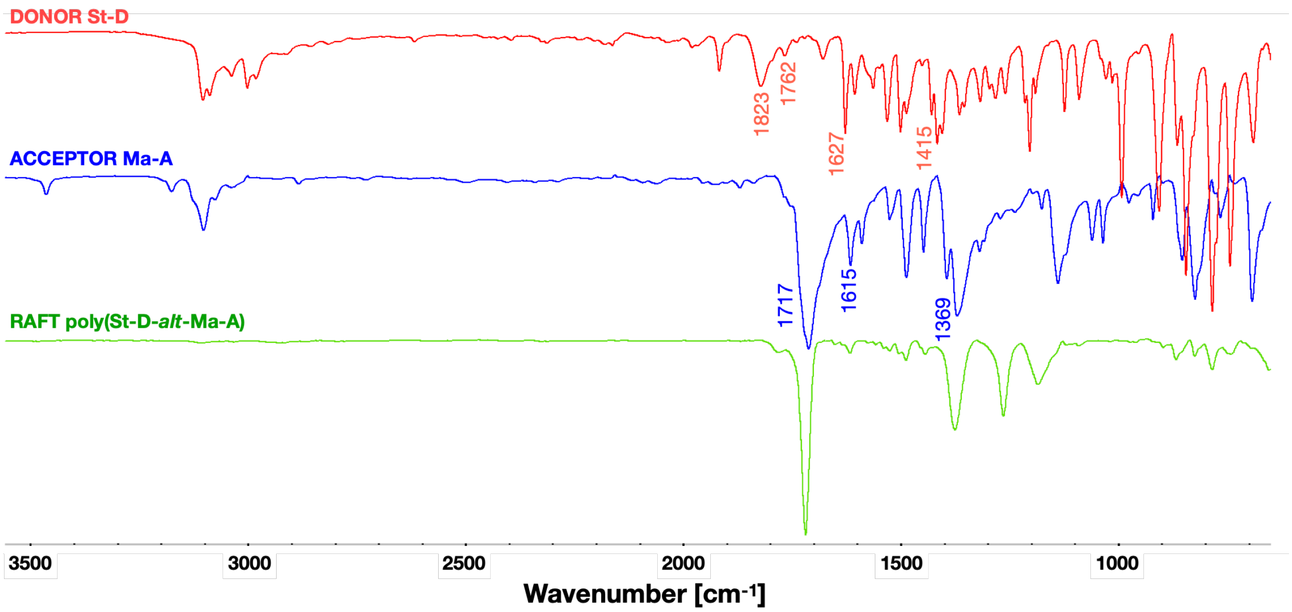

Figure S5. The IR transmittance spectra of synthesized donor and acceptor monomers, as well as the polymer brushes obtained via surface-initiated RAFT polymerization.

Donor (**St-D**): 1415 cm^{-1} (C=C stretching vibrations in thiophene ring), 1627 cm^{-1} (C=C stretching vibrations), 1823 and 1762 cm^{-1} (C=C overtones of stretching vibrations in benzene ring);

Acceptor (**Ma-A**): 1369 cm^{-1} (C=N stretching vibrations in benzo[c][1,2,5]thiadiazol ring), 1615 cm^{-1} (C=C stretching vibrations), 1717 cm^{-1} (C=O stretching vibrations in carbonyl group).

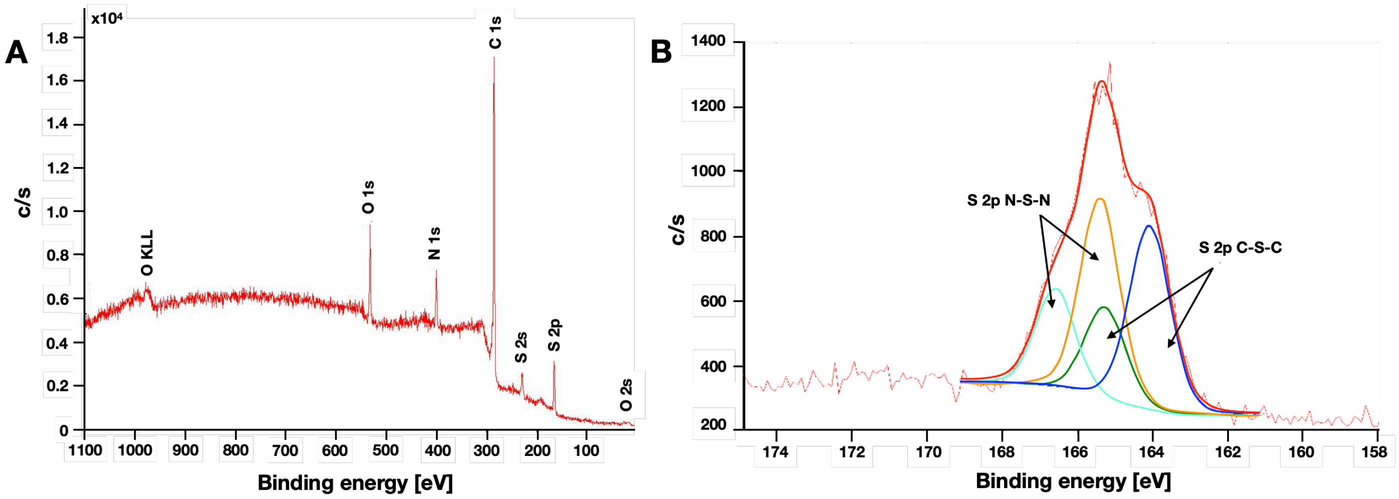

Figure S6. The XPS spectrum of (**A**) poly(**St-D**-*alt*-**Ma-A**) brushes obtained via RAFT polymerization and (**B**) sulfur region.

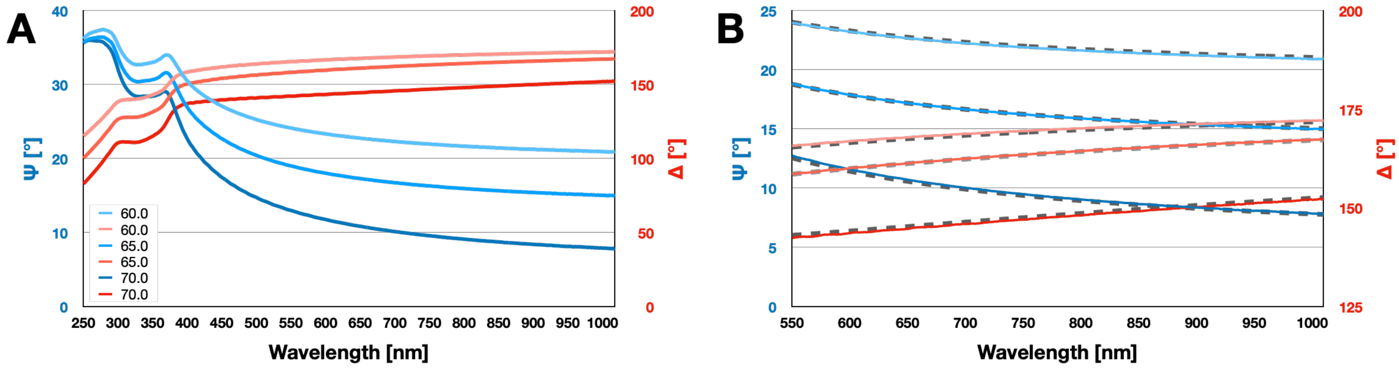

Figure S7. Exemplary spectroscopic ellipsometry data for the polymer brushes obtained via RAFT polymerization (**A**) and their fits to the Cauchy model (**B**).

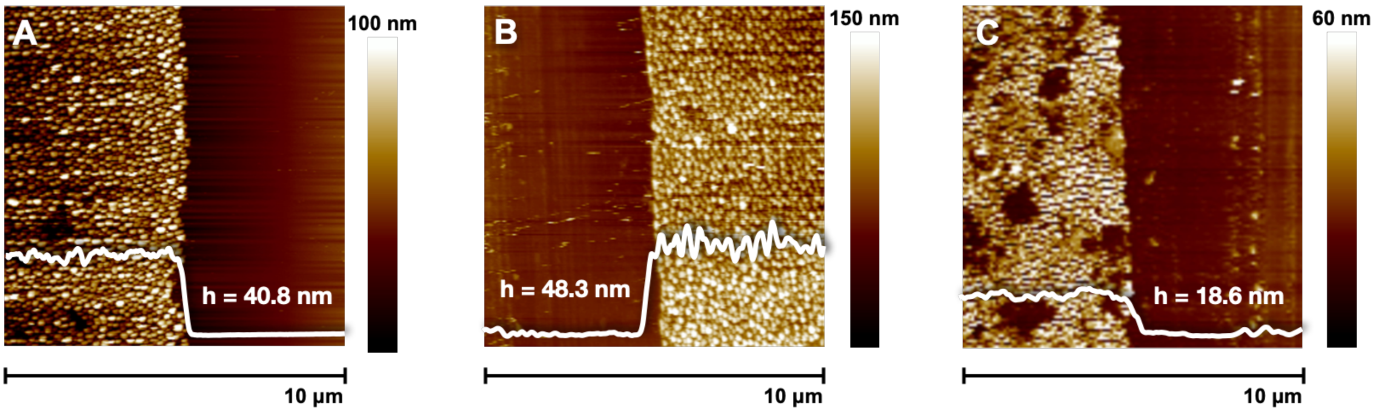

Figure S8. AFM pictures of poly(**St-D**-*alt*-**Ma-A**) brushes obtained via RAFT polymerization with various grafting densities: (**A**) 100% (**B**) 80% and (**C**) 70%.

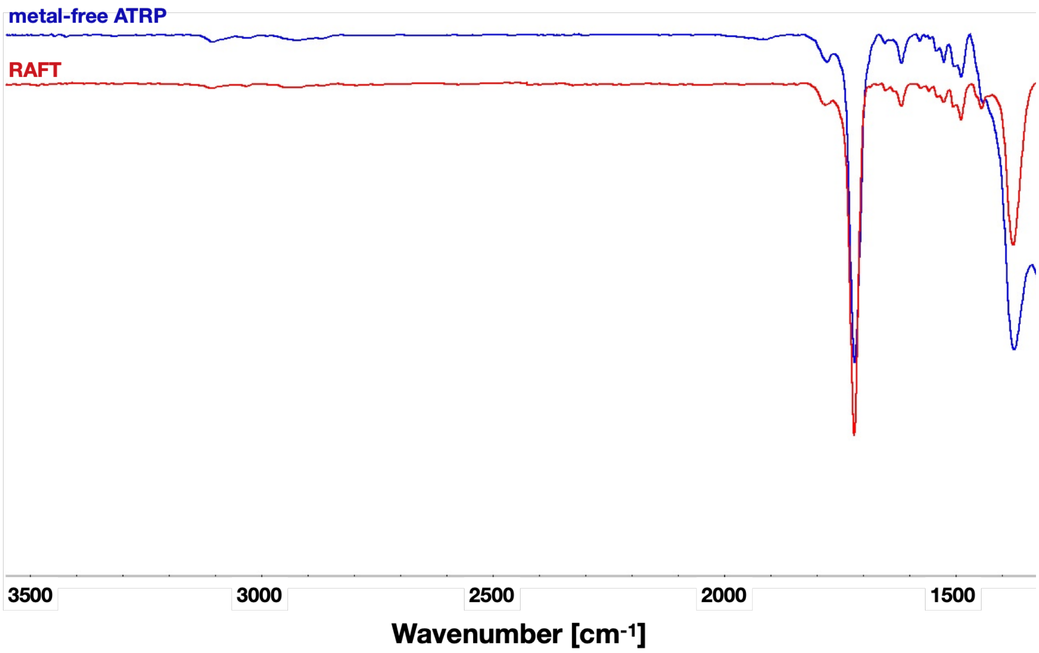

Figure S9. The IR transmittance spectra (1300 – 3500 cm^{-1}) of poly(**St-D**-*alt*-**Ma-A**) brushes obtained via RAFT and metal-free ATRP (the spectra at wavenumbers below 1300 cm^{-1} contain some artefacts related to the ITO substrates and are not comparable).

Table S1. Thickness of donor-acceptor brushes obtained during various time of metal-free ATRP polymerization

**Polymerization time [h]**	**Brush thickness based on spectroscopic ellipsometry [nm]**	**Brush thickness based on AFM [nm]**
0.75	3.5 ± 0.0	-
1.50	5.8 ± 0.1	5.1 ± 0.1
3.00	8.5 ± 0.1	9.8 ± 0.1
4.00	13.5 ± 1.2	13.4 ± 0.2
6.00	22.9 ± 0.3	26.9 ± 0.6

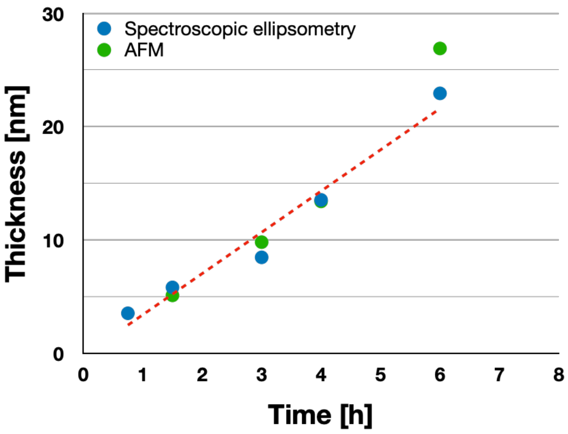

Figure S10. The relationship between thickness of donor-acceptor poly(**St-D**-*alt*-**Ma-A**) brushes and time of metal-free ATRP polymerization.

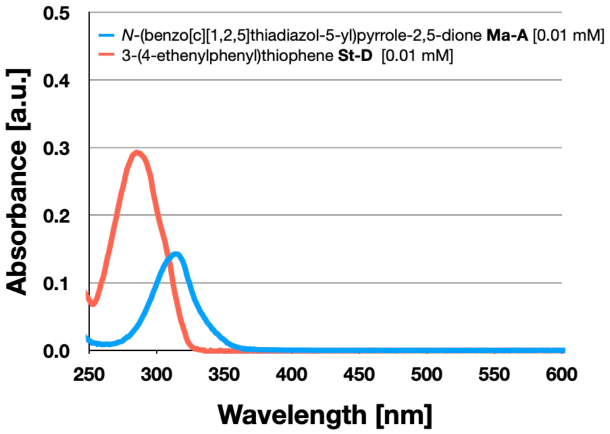

Figure S11. UV-Vis absorption spectra of the monomers used to synthesis of donor-acceptor polymer brushes. Solutions of both compounds are prepared in dioxane.
